# Tiger Nut Oil-Based Oil Gel: Preparation, Characterization, and Storage Stability

**DOI:** 10.3390/foods12224087

**Published:** 2023-11-10

**Authors:** Shanshan Zhang, Minghang Xin, Zhiyu Wang, Xiaolan Dong, Chenhe Yang, Hongcheng Liu, Hongxiu Fan, Tingting Liu, Dawei Wang

**Affiliations:** 1 School of Food Science and Engineering, Jilin Agricultural University, Changchun 130118, China; winkyshanshan@163.com (S.Z.); 15567288972@163.com (C.Y.);; 2 Engineering Research Center of Grain Deep-Processing and High-Effeciency Utilization of Jilin Province, Changchun 130118, China; 3 Scientific Research Base of Edible Mushroom Processing Technology Integration of Ministry of Agriculture and Rural Affairs, Changchun 130118, China; 4 Key Laboratory of Technological Innovations for Grain Deep-Processing and High-Effeciency Utilization of By-Products of Jilin Province, Changchun 130118, China

**Keywords:** tiger nut oil, *Cyperus esculentus* L., high internal phase emulsion, oleogel, stability research

## Abstract

In this study, Tiger nut (*Cyperus esculentus* L.) oil-based oleogels were prepared using the emulsion template method with whey protein (WPI; 0.5–2.5% (*w*/*v*) and Xanthan gum (XG; 0.1–0.5% (*w*/*v*). The microstructure of the oleogels obtained from the high internal phase emulsion (HIPE) and an emulsion after further shearing were observed using an optical microscope and laser confocal microscopy. A series of rheological tests were conducted to evaluate the effect of WPI and XG concentrations on the strength of the emulsion and oleogel. The texture, oil holding capacity, and oxidative stability of oleogels were characterized. The results showed that XG alone could not form oleogel, while the concentration of WPI had more effect than XG. When WPI was at a fixed concentration, the viscoelasticity of HIPE increased with the addition of XG. This was due to the complexation of WPI and XG, forming a stable gel network between the tight emulsion droplets and thus giving it a higher viscoelasticity. With an increase in WPI concentration, the stability and viscoelasticity of the emulsion were increased, and the oil-holding capacity and gel strength of the oleogels were enhanced. Moreover, the addition of XG could significantly enhance the stability and viscoelasticity of the emulsion (*p* < 0.05), and an increase in the concentration had a positive effect on it. The oleogels showed high gel strength (G′ > 15,000 Pa) and good thixotropic recovery when the XG concentration was higher than 0.3% (*w*/*v*). WPI (2.0%) and XG (>0.3%) could be used to obtain HIPE with good physicochemical and viscoelastic properties, which in turn lead to oleogels with minimal oil loss, viscoelastic and thixotropic recovery, and temperature stability. Compared with tiger nut oil-based oleogel, tiger nut oil contained more polyunsaturated fatty acids, which were more easily decomposed through oxidation during storage and had lower oxidation stability. This study provides a reference for the preparation of oleogels from food-approved polymers and provides additional theoretical support for their potential application as solid fat substitutes.

## 1. Introduction

Solid oils are an important component in the production of many foods because they play a critical role in changing or enhancing the texture and flavor of foods, emulsifying, improving crispness, fermenting, preventing sticking, transferring heat during frying, and providing a sense of fullness [1]. However, many solid oils contain a large amount of saturated fatty acids (SFA) and trans fatty acids (TFA). Regular consumption of foods with high levels of SFA and TFA increases the risk of cardiovascular disease, type II diabetes, and coronary heart disease [2]. The current consumer trend is to ensure health without altering the sensory flavor of food. Therefore, the development of new food-specific fat substitutes with low saturated fatty acids, high unsaturated fatty acids, and zero trans fatty acids is the focus of current research efforts [3].

Recently, there has been a growing interest in making oleogels from vegetable oils as an alternative to food-specific fats and oils. Structuring vegetable oils into oleogels as an alternative to food-specific fats and oils has received increasing attention. Oleogels have developed into a viable strategy for obtaining food-specific fats and oils. Oleogels are gel systems in which vegetable oils (>90%) are immobilized in a three-dimensional network structure in the presence of a gelling agent and exhibit the properties of solid fats and oils [4,5]. There are three main methods for preparing oleogels. Oleogels are mainly prepared by direct dispersion, indirect preparation, and physical adsorption. The direct dispersion method is to diffuse the gelling agent directly into liquid vegetable oil at a temperature exceeding the melting point of the gelling agent, mix it by stirring, and form an oleogel after cooling. The oleogel mechanism is divided into two kinds: the crystalline particle system of the gelling agent (biological wax [6], monoglycerides [7], fatty acids [7], fatty alcohols, phytosterols [8], etc.) and the molecular self-assembly structure (ethyl cellulose [9], ceramides [10], lecithin [10], 12-hydroxystearic acid [11], etc.). The indirect preparation method is the emulsion template method, which uses an amphiphilic (lipophilic and hydrophilic) gelling agent (protein, polysaccharide, and other high polymers) and edible vegetable oil to form an emulsion by mixing and homogenizing, and then the oleogel is prepared by shearing at room temperature or after freeze–drying [12]. The physical adsorption method is the foam template method, in which foam is formed by homogenizing and aerating the gel solution, freeze–drying it, adding edible vegetable oil, and shearing it to form an oleogel [13]. Compared with oleogels constructed using other methods, oleogel systems constructed using the emulsion template method are less affected by the external environment and have a more stable structure and oxidative stability [14]. Santiago et al. [15] used surface active polysaccharide (cellulose) and non-surface active polysaccharide (xanthan gum) as gels, and the prepared sunflower seed oil gel and olive oil gel made using the emulsion template method had good gel structure and oxidation stability.

Oleogels can form a unique stable network structure to convert edible vegetable oils from liquid to solid and retain the beneficial fatty acid components in vegetable oils and fats to a maximum extent, and these unique structural and lipid properties give them a greater potential for application in food processing [16]. Oleogels play a role in food processing mainly by replacing traditional artificial fats and oils (including SFA and TFA) and natural animal fats and oils (including SFA) with plasticity, controlling the flow and migration of liquid oils through the constraints of unique stabilizing network structures; carrying fat-soluble nutrients through unique stabilizing network structures; controlling and slowing the release of flavor substances. Specific applications in food processing include meat products, dairy products, bakery products, sauces, chocolate products, and nutraceuticals [17].

The presence of amino and carboxyl groups in protein molecules makes them surface active and capable of stabilizing oil–water emulsions [18]. Hydrophilic polysaccharides play an auxiliary role in stabilizing the emulsion template by increasing the viscosity of the continuous phase and forming an extended network. Previous studies have mostly prepared low internal phase emulsions, which require longer drying time in the preparation of oleogels, which leads to oxidation of oils and fats and increased energy loss. High internal phase emulsion (HIPE) is an emulsion with an internal phase volume fraction of 74.05% or more, which is more viscous and has a better gelation state than normal emulsions, and the use of HIPE as a template can reduce these problems [19]. The preparation of conventional HIPE requires a large amount of surfactants, and some surfactants are not allowed to be added or are amount limited in food [20].

Whey protein isolate (WPI) is a by-product of cheese production and is refined to obtain a protein with a protein content of more than 90%, which contains a variety of amino acids, has a reasonable composition, has good emulsification properties, and is a common stabilizer for emulsions [21]. The tertiary structure of proteins exposes more hydrophobic amino acid groups after heat treatment, which is more favorable for binding at the oil–water interface, and it has been shown that the viscosity and elasticity of stabilized emulsions of β-lactoglobulin increase after heating [22]. Xanthan gum (XG) is a natural microbial extracellular anionic polysaccharide with good solubility, gelation, and stability to heat and acids and bases, widely used in food, cosmetics, pharmaceuticals, etc. and has been studied in assisting the stabilization of emulsions. Recently, it has been mainly used for the preparation of emulsion template oleogels [4,23]. Espert et al. obtained stabilized solid-like oleogels using XG in combination with four different structuring agents [24].

*Cyperus esculentus* L., known as tiger nuts, underground walnuts, chestnuts, etc., is an annual herb of the salviaceae family, which is highly adaptable to the environment and is a new economic crop that integrates grain, oil, grazing, and feeding with high yield and high-quality, comprehensive utilization value. More interestingly, the oil content of tiger nut tubers is as high as 20–36%, which is higher than that of soybeans [25,26]. Tiger nut oil contains a large amount of unsaturated fatty acids, and the fatty acid composition and content are similar to those of olive oil and hazelnut oil. The unsaturated fatty acid content of tiger nut oil is generally above 85%, mainly oleic acid (65.5–76.1%), which has the effect of lowering cholesterol and preventing cardiovascular diseases, and it is considered to be a healthy vegetable oil [27,28]. Moreover, tiger nut oil is a major source of natural antioxidant components such as phytosterols, total phenols, tocopherols, and squalene, which have hypolipidemic and antioxidant effects [29]. In addition, the iodine value, specific gravity, viscosity, and energy content of tiger nut oil are comparable to sunflower oil, soybean oil, and canola oil [30]. Therefore, it is especially important to maintain the original quality of tiger nut oil for later applications.

In this research study, a tiger nut oil-based oleogel was prepared using the emulsion template method. WPI and XG solution were used to stabilize HIPE, and then HIPE was used as the template to construct the tiger nut oil-based oleogel. The particle size, rheological characteristics, macro and microscopic morphology, centrifugal stability, and oxidation stability, and, using the WPI and XG solution concentrations, the impacts on the physical and chemical properties and oxidation stability of tiger nut oil-based oleogels were explored. It provides a theoretical basis for the research and development of tiger nut oil-based oleogels and their replacement for food special oil.

## 2. Materials and Methods

### 2.1. Materials

Tiger nuts oils (TNO) were provided by Jilin Province Tiger Nuts Oils Agricultural Technology Co. (Changchun, Jilin Province, China). Whey protein isolate (WPI) from Hilmar Co. (St. Louis, MO, USA) contained 94.0% protein, 2.7% ash, 1.3% fat, and 0.1% lactose as dry matter. Nile Red, Nile Blue, and Fluorescent Whitening 28 were purchased from Sigma-Aldrich Co., Ltd. (Beijing, China). The other chemicals are of analytical grade.

### 2.2. Preparation of Stock Solution

According to the method of Ni et al. [31], WPI was dissolved in 100 mL distilled water, 0.02% (*w*/*v*) sodium azide was added, and the suspension was magnetically stirred at room temperature for 2 h. The suspension was refrigerated at 4 °C for 12 h to completely hydrate the WPI and the suspension was adjusted to pH = 7 using 0.1 mol/L NaOH, heated in a water bath at 85 °C for 30 min to completely denature the WPI. After the heat treatment, it was quickly cooled to room temperature to form a WPI stock solution with a concentration of 2.5% (*w*/*v*). The solution was stored for 24 h at 5 °C to ensure complete hydration of the biopolymer.

XG powders were weighed into 100 mL Milli-Q waters to make an XG stock solution with a concentration of 0.5% (*w*/*v*); this dispersion was stirred continuously at room temperature until complete dissolution was obtained. The solution was stored for 24 h at 5 °C to ensure complete hydration of the biopolymer.

### 2.3. Preparation of the High Internal Phase Emulsion (HIPE)

The WPI of the XG stock solution was diluted to the desired concentrations, added to tiger nuts oil, and homogenized for 3 min using a high-speed shear emulsifier (Fluko FM200, Shanghai, China) at 13,000 r/min to form HIPE [32].

Sample final system: Tiger nuts oil (TNO) phase volume fraction of 75%, WPI concentrations of 0, 0.5, 1.0, 1.5, 2.0, 2.5% (*w*/*v*) and XG concentrations of 0, 0.1, 0.2, 0.3, 0.4, 0.5% (*w*/*v*) HIPE were tested, while samples prepared with the same concentrations of XG or WPI were used as controls.

### 2.4. Preparation of Tiger Nuts Oleogel

According to the method of Wijaya et al. [33]. 30.0 g of HIPE was dried in the oven at 40 °C for 24 h to a constant weight to form a soft solid-like sample, which was processed by stirring and crushing and then sheared for 2 min using a shear emulsifier at 10,000 r/min to obtain the Tiger nuts oleogel, and the sample was stored at 5 °C.

### 2.5. Characterization of a High-Grade Internal-Phase Emulsion

#### 2.5.1. Determination of Particle Size and Distribution

Detection of particle size evaluates the stability of the emulsion. HIPE particle size was determined to determine the average particle size of emulsion droplets. Test conditions: At room temperature, the relative refractive indexes of the dispersed and continuous phases were 1.469 and 1.330, respectively, and a small amount of emulsion was diluted and dispersed in flowing distilled water (2400 r/min) to make the shading rate reach about 10%. The mean particle size of the emulsion is represented by the mean volume fractional diameter (D_[4,3]_) and is calculated using the following equation:

$$D_{\left[4,3\right]}=(\sum n_{i}d_{i}{}^{4}/\sum n_{i}d_{i}{}^{3})$$

where *n_i_* is the number of droplets in the *i*-size region; *d_i_* is the average particle size in the *i*-size region.

#### 2.5.2. Optical Microstructure of High-Grade Internal-Phase Emulsion

The microstructure of the prepared HIPE was visualized using a light microscope (DS-F13, Nikon, Tokyo, Japan). A drop of the solution was applied on a slide and covered with a coverslip. Subsequently, the samples were observed at 400× magnification using a digital microscope camera [34].

#### 2.5.3. Centrifugal Stability

According to the method of Yan et al., fresh samples were centrifuged at 25× *g* at 8000 r/min for 20 min to observe the emulsion separation and were photographed immediately [35].

#### 2.5.4. Rheological Properties Measurements

According to the method of Zhang et al. with modifications, the rheological properties of HIPE samples were determined using a 40 mm parallel plate of a DHR-1 rheometer (DHR-1, TA Instruments, New Castle, DE, USA). Static apparent viscosity is determined in the shear rate range of 0.1 s^−1^ to 100 s^−1^. Frequency sweep measurements (frequency scan range of 0.1 Hz to 10 Hz, strain determined as 1%) to determine the energy storage modulus G′ and loss modulus G″ of the HIPE samples. All measurements were performed at 25 °C [36].

### 2.6. Characterization of the Tiger Nut Oil-Based Oleogel

#### 2.6.1. Rheological Properties

The apparent viscosity change in the oleogels samples was determined using a 40 mm plate of a DHR-1 rheometer (DHR-1, TA Instruments, New Castle, DE, USA) with a shear rate range of 0.1 s^−1^ to 100 s^−1^ and by frequency scan (frequency scan range of 0.1 Hz to 10 Hz, strain determined as 1%) to determine the energy storage modulus G′ and loss modulus G″ of the HIPE samples, both at 25 °C [36].

The shear viscosity and frequency scanning analysis of the oleogels were modified according to the method of Meng et al. [37]. A time scan was used to measure the recovery ability of the sample at alternating shear rates (0.1 s^−1^, 10 s^−1^, 0.1 s^−1^) for 300 s. The temperature scan was used to measure the sensitivity of the sample at temperature test from 5 to 80 to 5 °C.

#### 2.6.2. Optical Microstructure

Freshly prepared equal amounts of HIPE were loaded into sample bottles, observed, photographed, and recorded for macroscopic evaluation. The microstructure was further observed using an optical microscope, a small number of samples were taken on the slide, and the cover slide was gently flattened and photographed at 400 (eyepiece 10, objective 40) [34].

#### 2.6.3. Determination of Oil Loss

Oil loss (OL) in oleogels was determined using centrifugation according to the method of Meng et al. with modifications [37]. The centrifuge tube was dried in the oven to constant weight ($m_{0}$), a 4.0 g oleogel sample was weighed and centrifuged for 20 min at 9000 r/min, the free oil was poured through a filter paper and drained for 20 min, and the total weight of the remaining gel oil sample and centrifuge tube (m) were measured. The calculation formula is:

$$\mathrm{OL}=\frac{m-m_{0}}{M-m_{0}}\times 100\%$$

where m is the total weight of the remaining gel oil samples and centrifuge tubes; m_0_ is the weight of the centrifuge tube; M is the weight of the oil–gel sample.

#### 2.6.4. Confocal Laser Scanning Microscopy (CLSM)

The microstructure of oleogels was observed using confocal laser scanning microscopy (CLSM). The microstructure was slightly modified according to the method of Bascuas et al. [38], and the microstructure of the samples was observed using a laser confocal microscope SP8 (Carl Zeiss Microsystems, Mannheim, Germany) with a scanning mode of 512 × 512 pixels; scanning frequency of 400 Hz, staining of grease using Nile Red with an excitation wavelength of 514 nm; staining of XG using Fluorescence Brightening 28 with an excitation wavelength of 405 nm; and staining of WPI using Nile Blue A small amount of sample was placed on the slide, and 20 μL of Nile Red, Fluorescent Brightening 28 and Nile Blue were added to the slide, and the sample was stained for 30 min, covered with a coverslip to observe and record the microstructure.

#### 2.6.5. Oxidative Stability

The oxidative stability of the oleogels was evaluated with an oil oxidation analyzer (Oxitest, VELP Co., Lombardia, Italy). It was to characterize the oxidative stability of the product in terms of oxidation induction time [39]. A mass of 5.00 g of tiger nuts oil and tiger nuts-based oleogels were weighed in a sample tray, and a sealing ring was used to seal them. It was measured at a constant temperature of 90 °C and a reaction chamber oxygen pressure of 6 Bar. The oxidation induction time used was the software that came with the instrument.

#### 2.6.6. Fatty Acid Composition

The tiger nut oil and oleogel were placed in an incubator at 50 °C, and the fatty acid composition of the samples was determined and analyzed on day 1 and day 30.

The methyl esterification of the sample was determined by the method of ISO 12966-2:2017. The fatty acid composition was studied using a Thermo Fisher TSQ9000 gas chromatograph (TSQ9000; Thermo Fisher Scientific, Waltham, MA, USA). Firstly, the preparation of fatty acid methyl esters was carried out by saponifying the oleogel with 0.5 M KOH, followed by methylation with 40% boron trifluoride in methanol. The chromatographic conditions were as follows: chromatographic column: HP-88 capillary column (100 m × 0.25 mm × 0.20 μm); heating program: 125 °C kept for 0.5 min, then increased to 145 °C at 10 °C/min, then to 180 °C at 5 °C/min, kept for 15 min, and to 230 °C at 5 °C/min; carrier gas was high-purity helium at a flow rate of 1.0 mL/min. The carrier gas was high-purity helium with a flow rate of 1.0 mL/min, a split ratio of 50:1, a sample volume of 1 μL, and an inlet temperature of 250 °C.

Mass spectrometry conditions: electron bombardment (EI) ion source; electron energy of 70 eV; transfer line temperature of 250 °C; ion source temperature of 230 °C; solvent delay of 6 min; mass scanning range of 40~600 *m*/*z*.

### 2.7. Statistical Analysis

These experimental data are expressed as the mean of triplicate experiments. The significance was analyzed using a one-way analysis of variance (ANOVA) and Duncan-test in SPSS 22.0. *p* < 0.05 was the statistically significant difference and graphed using Origin 2022 software.

## 3. Results

### 3.1. The Particle Size and Size Distribution of HIPE

**Figure 1:**
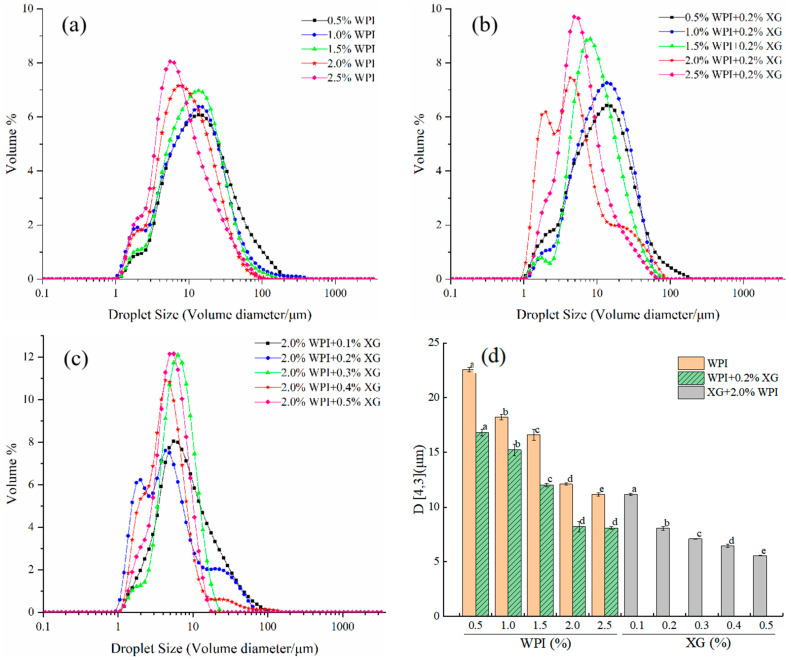
Droplet size distribution curve of emulsion prepared for HIPE with different concentrations of WPI and XG and volume-weighted average droplet size (D4,3). (**a**) HIPE of WPI. (**b**) HIPE of (WPI+0.2%XG). (**c**) HIPE of (2.0%WPI+XG). (**d**) (D4,3) of HIPE with different concentrations of WPI and XG. Different lowercase letters(a–d) in figure (**d**) represent significant differences (*p* < 0.05).

The particle size and size distribution of emulsion droplets are key indicators of the stability of emulsions. As can be seen in Figure 1a,d, the size distribution of HIPE droplets stabilized by WPI alone (0.5–2.5% *w*/*v*) was in the range of 0.92–255.58 μm. However, as the WPI concentration increased, the particle size distribution curves were all single-peaked (higher peaks) and all moving toward a smaller direction. D4,3 gradually decreased between 22.57 and 11.17 μm, and the difference between the concentrations was significant (*p* < 0.05). This might be because an increase in WPI concentration made the interfacial adsorption of emulsion droplets increase, resulting in a decrease in interfacial tension, making the emulsion particle size smaller [40]. From Figure 1b,d, it can be seen that the HIPE droplet size distribution of 0.2% *w*/*v* XG + WPI (0.5–2.5% *w*/*v*) showed a similar trend to WPI alone, with a narrower distribution range of 0.93–204.95 μm. The D4,3 was 16.83–8.10 μm, and the addition of XG made the D4,3 of HIPE significantly smaller, which was consistent with the results of Hu Yu et al. [41]. As can be seen from Figure 1c,d, the droplet size distribution of HIPE with 2% WPI + XG (0.1–0.5% *w*/*v*) changed from a trend in the volume particle size distribution curve with increasing XG concentration in general agreement with the WPI. D4,3 decreased with increasing XG between 11.17 μm and 5.56 μm, and D4,3 was (7.10 ± 0.02), (6.46 ± 0.17), and (5.56 ± 0.02) μm for XG concentrations between 0.3% and 0.5%, respectively. When the relative XG concentration was 0.1~0.2%, a similar phenomenon was found in a study conducted by Patel et al. [23].

### 3.2. Macroscopic and Microstates of HIPE

**Figure 2:**
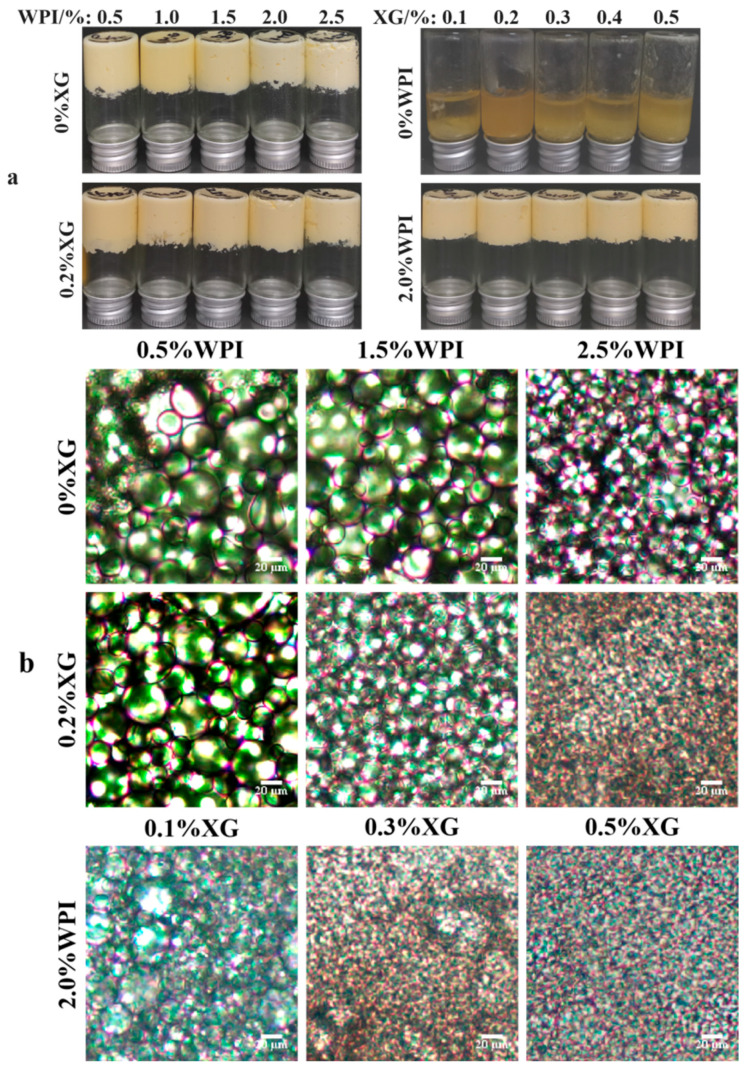
The macro (**a**) and micromorphology (**b**) for HIPE with different concentrations of WPI and XG.

The appearance of HIPE formed by different concentrations of WPI and XG is shown in Figure 2a. It can be seen that the sample stabilized by only XG could not form HIPE at all test concentrations, while the HIPE stabilized by WPI, or WPI and XG together, could form a complete system under all test conditions and did not flow upside down, which has strong stability [23]. Figure 2b shows the microstructure of HIPE stabilized by different concentrations of WPI and XG. With an increase in WPI concentration, the emulsion droplet size gradually became smaller, and the emulsion droplets were uniformly distributed throughout the system. The emulsion droplet size with the addition of 0.2% XG was significantly smaller than that of HIPE stabilized by WPI alone, and the emulsion droplets were more tightly distributed, which was consistent with the results of the previous particle size study. This result was in line with a report by Liu et al. (2021) [42]. The emulsion droplet size of HIPE co-stabilized by 2.0% WPI and 0.1% XG was significantly smaller than that of HIPE co-stabilized by 0.5% WPI and 0.2% XG, and the local oil leakage that occurred was probably due to the excessive production force during optical microscopy observation. The droplet size of the HIPE co-stabilized by 2.0% WPI and 0.5% XG was the smallest, as seen from all microstructure maps, and this phenomenon can also be seen in D4,3 data.

### 3.3. Rheological Properties of the HIPE

**Figure 3:**
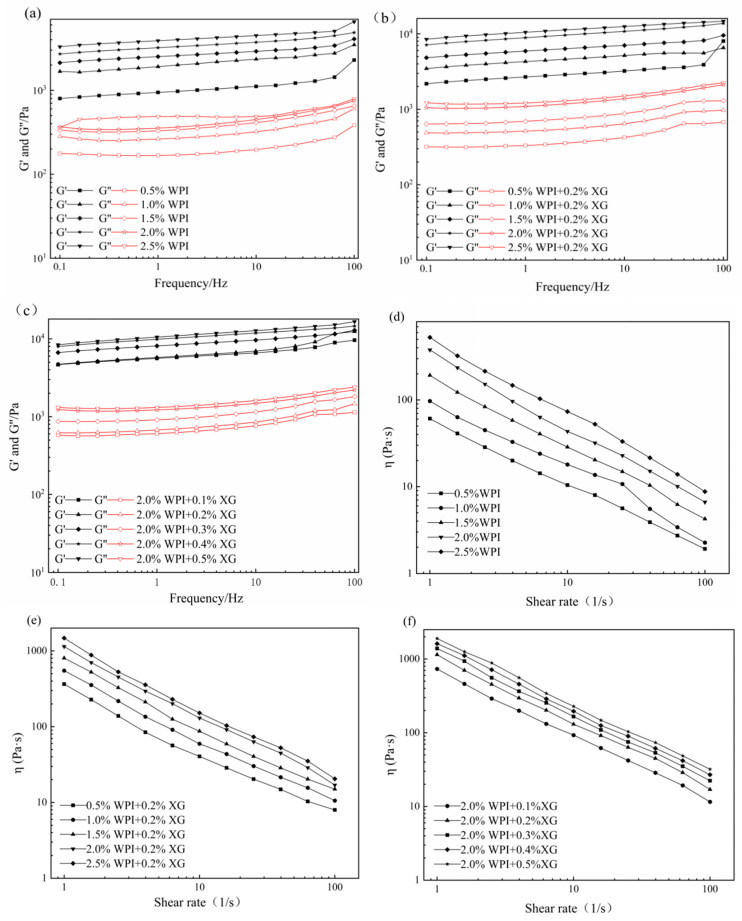
Frequency sweep (**a**–**c**) and shear viscosity(**d**–**f**) curves for HIPE by different concentrations of WPI and XG.

The effects of WPI and XG concentration changes on the elastic modulus (G′) and viscous modulus (G″) of HIPE are shown in Figure 3. G′ of all samples was much higher than the corresponding G″, indicating that the HIPE stabilized by WPI, or WPI and XG together, had elastic gel behavior (Figure 3a,b). In the frequency range tested, both G′ and G″ values showed a weak dependence on frequency, and no intersection of the G′ and G″ values was observed, proving that the structure was not disrupted and no phase transition occurred (gel-sol) [43,44]. The values of G′ and G″ were obviously increased after adding 0.2% XG at equivalent WPI concentration, which indicated that the addition of XG could improve the viscoelasticity of HIPE. This result was similar to a report by Liu et al. (2021) [42]. It could be due to the complexation of WPI and XG to form a stable gel network between tight emulsion droplets, resulting in higher viscoelasticity [41]. In the frequency range of the experiment, both G′ and G″ increased with the frequency, and G′ was much higher than the corresponding G″, with the same trend as in Figure 3a,b. The difference between G’ of HIPE stabilized by high concentrations of XG (0.4% and 0.5%) was smaller and did not change as much as the effect of changes in WPI concentration, indicating that higher concentrations of XG could not cause a large increase in HIPE rigidity [45].

The apparent viscosity of all samples decreased with increasing shear rate, showing pseudoplastic behavior and the properties of a non-Newtonian fluid (Figure 3d–f). Moreover, the apparent viscosity was proportional to the WPI concentration at the same shear rate, which might be due to the smaller emulsion droplet particle size and tighter arrangement between emulsion droplets as the WPI concentration increased, which increased the shear resistance. The addition of XG resulted in a significant increase in apparent viscosity compared with WPI-stabilized HIPE alone, which may be attributed to the fact that the addition of XG increased the viscosity of the continuous phase and the interactions between emulsion droplets [46]. In addition, HIPE with XG addition was less affected by the shear rate, indicating that XG could enhance the stability of HIPE. As shown in Figure 3f, the apparent viscosity of HIPE with XG concentrations was similar to that of Figure 3d,e, exhibiting the pseudoplastic behavior of non-Newtonian fluids. Furthermore, the apparent viscosity increased with increasing XG concentration at the same shear rate.

### 3.4. The Centrifugal Stability Analysis of the HIPE

**Figure 4:**
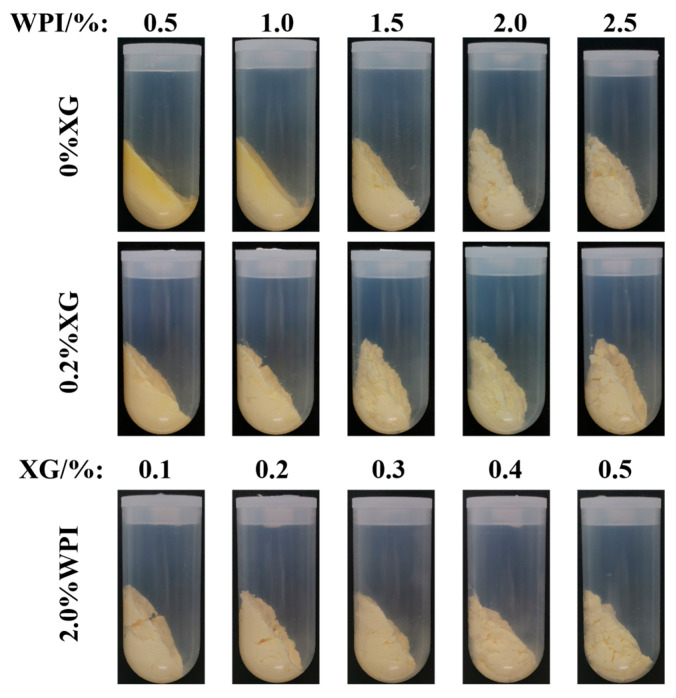
Stability of HIPE after centrifugation with different concentrations of WPI and XG.

Centrifugal stability tests were used to evaluate the effect of WPI and XG concentrations on the stability of HIPE, which could be used to demonstrate the strength of the gel network structure. Only a strong enough gel network structure can form a stable HIPE, which is a prerequisite for the preparation of physically stable oleogels [41]. As can be seen in Figure 4, after high-speed centrifugation, only HIPE stabilized by lower concentrations of WPI (0.5% and 1.0%) had less water appearing, and a yellow part appeared in the upper layer of the emulsion, considering the occurrence of oil leakage. No moisture and oil leakage was found after the addition of 0.2% XG, indicating that the addition of XG enhanced the gel network structure and was able to resist deformation during centrifugation. XG added to emulsions markedly affects oleogel stability [14]. This might be due to the formation of colloidal complexes of XG and WPI forming the inner and outer layers of encapsulated oil droplets in the HIPE system. In addition, the appearance morphology of HIPE became denser and thicker as the concentration of WPI and XG increased.

### 3.5. Rheological Properties of the Tiger Nut Oil-Based Oleogel

**Figure 5:**
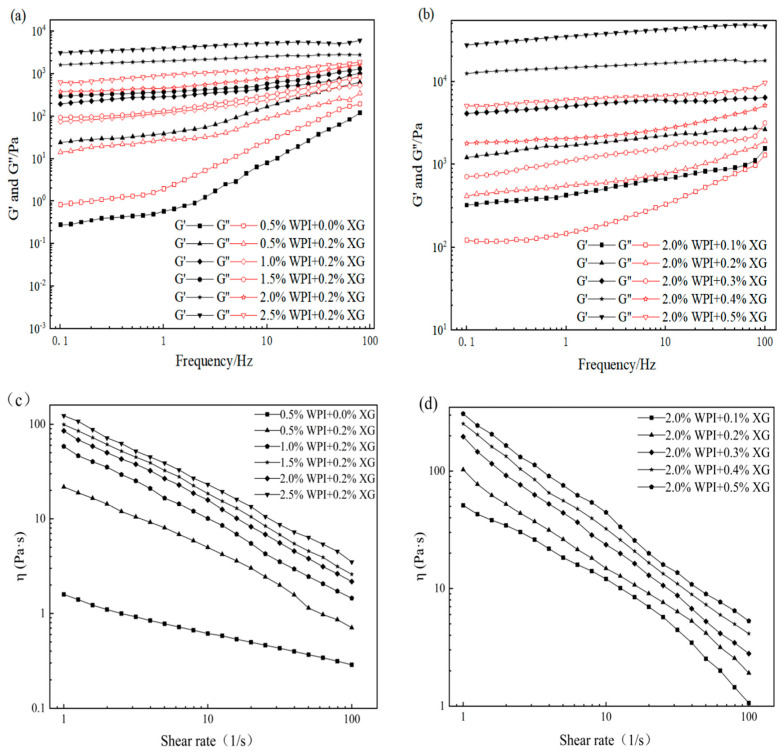
Frequency sweep(**a**,**b**) and shear viscosity (**c**,**d**) curves of oleogels obtained via templating of HIPEs stabilized by different concentrations of WPI and XG.

The viscoelasticity and apparent viscosity of oleogels are influenced by the polymer type and concentration of the constructed oleogels [23,47]. The rheological properties of the oil gel samples prepared with different concentrations of XG and WPI are shown in Figure 5. As can be seen in Figure 5a,b, all the oleogels exhibit solid behavior (G′ > G″) throughout the frequency range, except for the oleogel sample stabilized by 0.5% WPI alone. The oleogels stabilized by 0.5% WPI alone underwent mainly viscous deformation and exhibited liquid mobility (G″ > G′) (Figure 5). It can be seen from Figure 5 that the addition of XG caused a phase transition from liquid fluidity to elastic gel properties, the dependence of G′ and G″ on frequency gradually became smaller, and the viscoelasticity increased with an increase in WPI concentration, indicating an increase in gel strength. The mechanical strength of the oleogels was more significantly affected by the XG concentration under the condition of a fixed WPI concentration (Figure 5b). In contrast, an increase in WPI concentration had less effect on the mechanical strength of the oleogels (Figure 5a). The values of G′ and G″ showed low-frequency dependence at all frequencies from 0.1 to 100 Hz. Interestingly, none of the curves showed a crossover point (G′ = G″), indicating that the oleogel did not transform from a gel to a sol even at higher frequencies [23]. Moreover, XG concentration has an obvious effect on the viscoelasticity of oleogels because the network structure originally supported in HIPE is attached to the surface of oil droplets after HIPE drying, and the viscoelasticity increases as the XG concentration increases [48]. Figure 5c,d showed that all the oleogels showed shear thinning, along with WPI and XG concentration increasing, its apparent viscosity also increased, and the apparent viscosity of samples without XG was significantly lower, with less effect on high concentrations of XG (0.3~0.5%). Therefore, the presence of XG and the concentration of WPI and XG in a sample can affect the viscoelastic energy and apparent viscosity.

**Figure 6:**
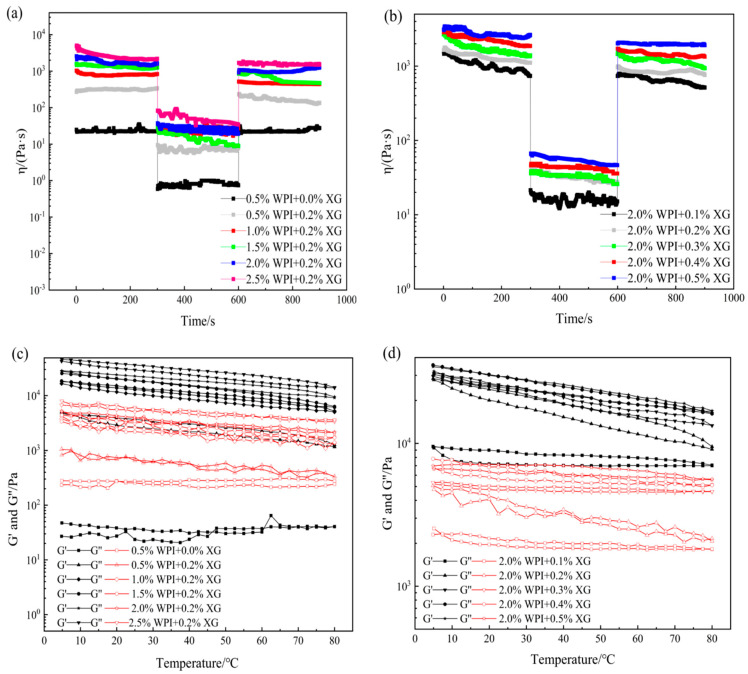
Time sweep (**a**,**b**) and temperature sweep (**c**,**d**) curves of oleogels obtained via templating of HIPEs stabilized by different concentrations of WPI and XG.

In actual processing applications, oleogels mainly depends on their touch recovery ability and thermal stability energy [49]. Temporal and temperature scans were performed for different WPI and XG concentrations. From Figure 6a,b, it can be seen that the viscosity of all the oleogel samples, except the sample stabilized by 0.5% WPI alone, showed a decreasing trend with time when the shear rate was constant, indicating that the change in sample viscosity was not only related to the shear rate but also to the shear time. When the shear rate changes rapidly (0.1 to 10 s^−^^1^), the viscosity drops immediately, which means that a large enough force would break the connection between the oleogel particles, leading to a reduction in their resistance to flow. The tactile resilience was characterized as the ratio of the apparent viscosity at the third shear rate to the first shear rate [50]. The samples stabilized by 0.5% WPI alone showed a smaller degree of apparent viscosity change with increasing time over the entire time scan range, exhibiting a strong thixotropic recovery. This might be due to the fact that the stabilizer did not form a more reticular structure inside it to protect the grease, making it close to the liquid oil state. However, the thixotropic response of the remaining groups of oleogels decreased slowly, and the thixotropic recovery ranged from 70 to 80%. It showed that oleogels had strong structural restoration properties in the resting state, which had a guiding meaning for the application of oleogels as fat substitutes in the baking, dairy, and meat products industries.

As can be seen from Figure 6c,d, throughout the temperature range, the samples showed elastic gel characteristics (except for the samples stabilized by WPI alone), which did not change with the temperature, indicating that the internal network structure is well maintained and showed stability with regards to temperature. With a constant XG concentration, G′ and G″ basically recovered during cooling, indicating that the temperature was in the range of 5 °C to 80 °C and did not cause damage to the internal structure of the oleogel. With constant WPI concentration and changing XG concentration, G′ and G″ recovered well during the cooling process. Stability may be due to the insensitive of XG to temperature and the irreversible change in WPI after thermal denaturation. Therefore, temperature does not greatly affect the formed structure of WPI at the temperature range from 5 to 80 °C. In summary, WPI and XG co-stabilized HIPE template oleogels at certain concentrations can exhibit good thixotropic recovery and temperature stability, which will lead to a wider application of oleogels in food (e.g., baking industry).

### 3.6. Macroscopic of the Tiger Nut Oil-Based Oleogel

**Figure 7:**
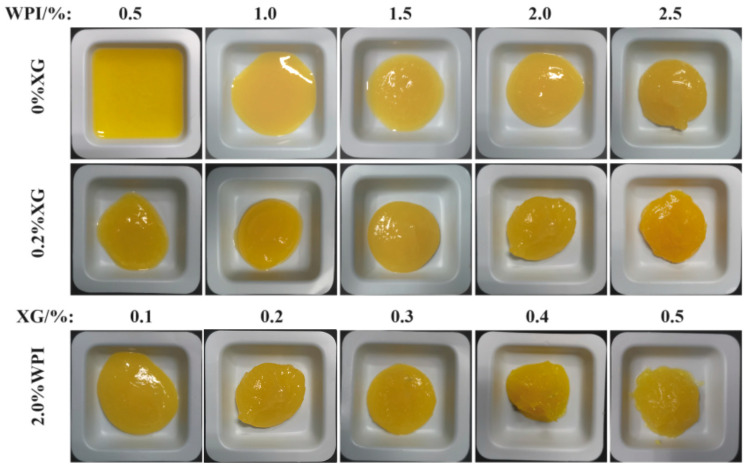
The macromorphology of oleogels obtained via templating of HIPEs stabilized by different concentrations of WPI and XG.

HIPE template oil gel macroscopic and microstates are shown in Figure 7. The macroscopic and microscopic states of the HIPE template oleogel are shown in Figure 7. At higher WPI or XG concentrations, the oleogels had a more solid-like appearance, had better formability, leaked little or no oil, and showed a positive correlation with concentration. Based on the macroscopic view of oleogels, only oleogels formed by WPI showed liquid behavior at low concentrations (<1.0%), the same as the rheological results, and the morphology and oil leakage improved with an increase in concentration. When XG (0.2%) was added, the solid behavior of the oleogels was strengthened with the concentration of WPI. When the WPI concentration was fixed at 2.0%, the addition of XG (0.2–0.5%) resulted in a better solidification of the oleogel, which exhibited a butter-like solid behavior and essentially no oil leakage. XG greatly improved the oil leakage from the oleogels compared to stabilizing the HIPE template oleogels by WPI only, which was consistent with the trend of the HIPE study results.

### 3.7. Stability of the Tiger Nut Oil-Based Oleogel

**Figure 8:**
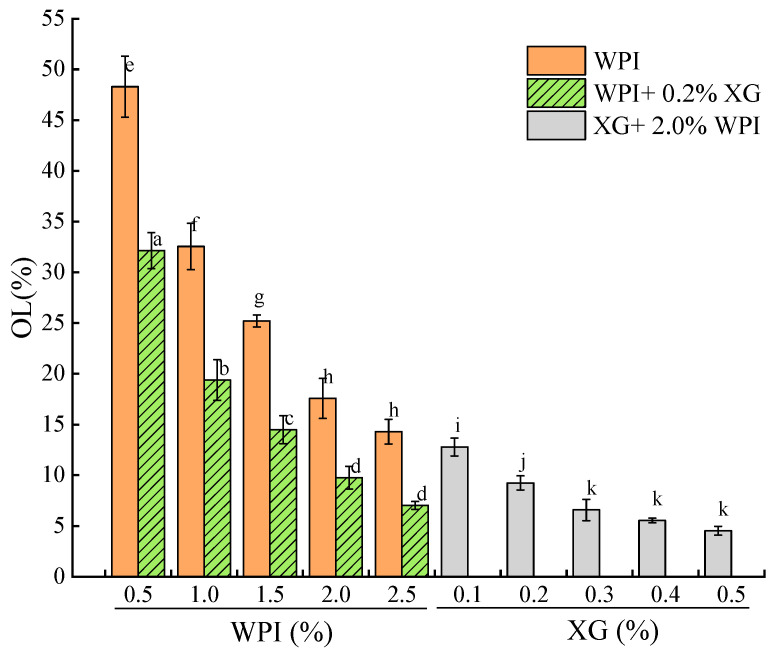
Oil loss of oleogels obtained via templating of HIPEs stabilized by different concentrations of WPI and XG. Different lowercase letters(a–d), (e–h), (i–k) represent significant differences (*p* < 0.05).

The OL of the oleogel indicates its stability, with a lower OL indicating a higher oil-holding capacity of the oleogel sample [51,52]. Figure 8 showed that the OL of the HIPE template oleogels stabilized by WPI alone was inversely proportional to WPI concentration, and the network structure formed at lower WPI was not sufficient to hold oil. In contrast, the OL of the samples spiked with XG was significantly lower. When the XG concentration was fixed at 0.2%, at lower WPI concentrations (0.5% to 1.5%), although the HIPE was stable, while the OL of the oleogel was larger between (14.49 ± 1.38)% and (32.14 ± 1.77)%, which might be due to the drying process breaking the interfacial membrane or the interfacial membrane was not strong enough and destroyed most of the structure. The oleogels exhibited lower oil leakage at WPI concentrations greater than 1.5%. When WPI was fixed at 2.0%, there was a difference (*p* < 0.05) in OL at higher XG concentrations (0.3–0.5%), with the smallest OL at 0.5% XG concentration. This indicates that the addition of XG significantly reduces OL and that OL stabilizes at high XG concentrations, which is consistent with the results of a related study [52].

### 3.8. Microstructure of the Tiger Nut Oil-Based Oleogel

**Figure 9:**
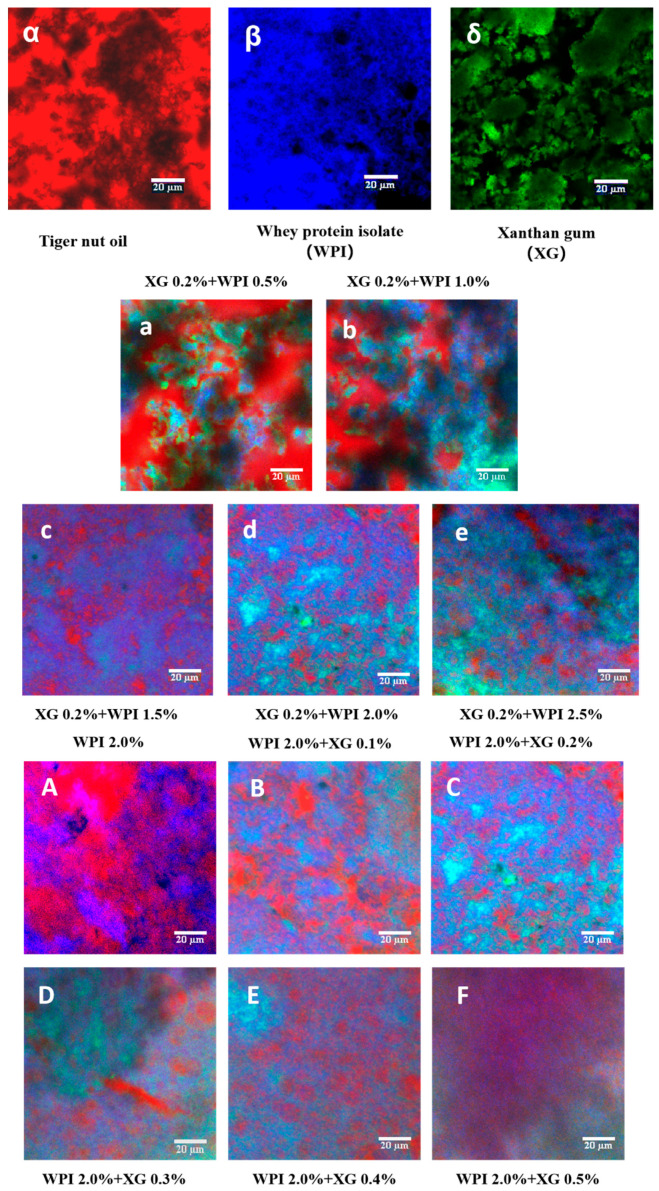
CLSM images of WPI-XG stabilized oleogel. (**α**–**δ**) are tiger nut oil, whey isolate protein (WPI), and xanthan gum (XG), respectively; (**a**–**e**) are 0.2 + 0.5, 0.2 + 1.0, 0.2 + 1.5, 0.2 + 2.0, and 0.2 + 2.5% (*w*/*v*) XG-WPI-stabilized tiger nut oil-based oleogels, respectively; (**A**–**F**) are 2.0 + 0.0, 2.0 + 0.1, 2.0 + 0.2, 2.0 + 0.3, 2.0 + 0.4 and 2.0 + 0.5% (*w*/*v*) of WPI-XG stabilized tiger nut oil-based oleogels; scale bars are 20 μm.

Figure 9 illustrates the laser confocal micrographs (CLSM) of tiger nut oil-based oleogels, in which XG, WPI, and HIPE template oleogels were labeled with fluorescent dyes to observe the microstructure of the samples. As shown in Figure 9α–δ, the XG fluorescence image is green, the WPI fluorescence image is blue, and the oil fluorescence image is red. From Figure 9a–e), it can be seen that at a fixed XG concentration of 0.2%, the dispersion of the red color in the oil phase becomes higher and higher with an increase in WPI concentration, and the area of the red color becomes smaller. The network structure formed by WPI and XG partially wraps around the oil droplets, which reduces the leakage of the oil. It is noteworthy that the encapsulation of oil in the XG–WPI gel system was enhanced when the concentration of WPI reached 2.0% (*w*/*v*) when the oil gel system was more stable. Therefore, the oil gel systems of XG0.2% + WPI2.0% and XG0.2% + WPI2.5% were more stable. In contrast, only the WPI-stabilized HIPE template oleogel showed a wide distribution of oils (Figure 9A); however, its network structure could only accommodate a fraction of these oils, which led to a large amount of leakage—consistent with the macroscopic observation in Figure 5. As the XG concentration increased (Figure 9B–F), more oils were seen to be uniformly encapsulated in this composite system through endosmosis between the networks formed by the XG and WPI gels, as well as an improved aggregation behavior compared with the individual components—this was particularly evident in the HIPE template colloids stabilized at XG concentrations above 0.2% *w*/*v* (Figure 9C–F. This finding is in agreement with the study of Bascuas et al. [34], who also confirmed that the oils were entrapped in a mesh structure formed by the stabilizer.

### 3.9. Oxitest Was Used to Evaluate the Oxidation Stability of Tiger Nuts-Based Oleogels

**Figure 10:**
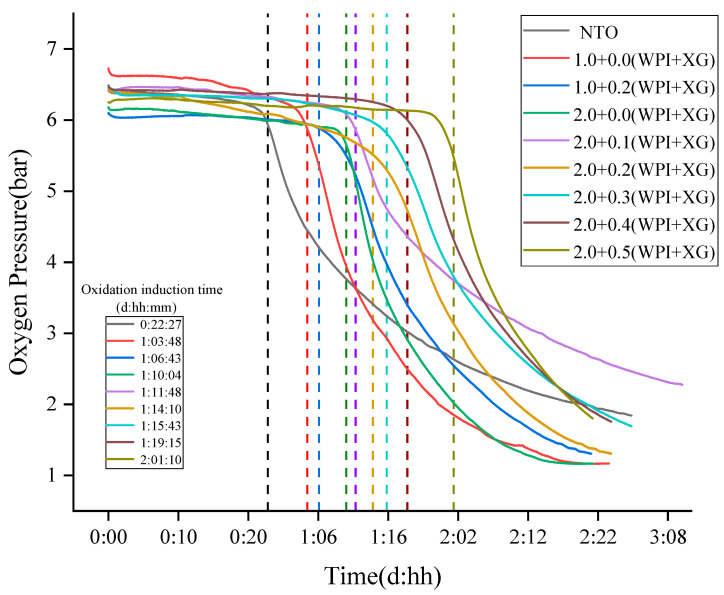
The Oxidation Stability of Tiger Nuts-Based Oleogels (The intersection of the colored dotted lines and the curves are the oxidation induction time).

The slow auto-oxidation of oil occurs at room temperature, which seriously affects its nutritional value. In particular, oils rich in unsaturated fatty acids, such as tiger nuts oil (NTO), are more susceptible to oxidation and deterioration. Oxitests are used to characterize the oxidative stability of a product in terms of oxidation induction time [53]. The principle is that under the condition of high-pressure oxygen, a high temperature is used to induce an oxidation reaction between food and oxygen to occur quickly. With the oxidation reaction, the oxygen pressure gradually decreases, and then the oxidation induction time of food is calculated [54]. First, the sample oxidation induction time was calculated at 90 °C, 6 bar, and 5 g of sample volume (measured by the mass of oil in the sample). According to the principle of calculating the induction time by the instrument, it was known that two tangents were made within the initial smooth phase and the rapid decline phase, and the intersection point was the induction time. The ability of the sample to react with oxygen determines the oxidation induction time of the sample, and the longer the oxidation induction time, the better the oxidation stability. The results are shown in Figure 10, and the oxidation induction time of tiger nut oils was 22:27 (hh:mm). Compared with tiger nut oil (NTO), the oxidation induction time of all oleogels samples was prolonged, indicating that the oxidation stability of tiger nuts-based oleogels was higher than that of NTO. When the WPI concentration was constant, the oxidation induction times of the oleogel samples were in the range of 001:10:04–002:01:10 (ddd:hh:mm), indicating that the addition of XG prolonged the oxidative stability of the oleogels. When the XG concentration was 0.5%, the oleogel samples showed the longest oxidation induction time (002:01:10) and the best oxidation stability. When the XG concentration was constant (0% or 0.2%), the addition of WPI increased the oxidation induction time of the samples. It is possible that the oxidative denaturation of WPI at high temperatures formed a gel structure, which prevented the oxidation of the liquid oil by oxygen. All these results indicated that the oleogelation of the NTO with WPI and XG caused an oxidative inhibition of the liquid oil. The good oxidative stability of the oleogel might be due to the fact that the liquid oil was sandwiched into the gel structure formed by WPI and XG, thus delaying the oxidation [55,56].

### 3.10. Fatty Acid Composition of Tiger Nuts-Based Oleogels

As we know, the fatty acid composition of fats and oils determines oxidative stability and nutritional properties. Fats and oils with a high content of unsaturated fatty acids are more susceptible to oxidative rancidity than those with saturated fatty acids, mainly because unsaturated fatty acids with an unstable carbon-carbon double bond are easily oxidized to a single bond, which reduces the oxidative stability of the fats and oils [57]. The fatty acid composition of tiger nut oil-based oleogel is shown in Table 1, where the oleogel and tiger nut oil (control) have similar fatty acid composition. From Table 1, it can be seen that the major fatty acids in tiger nut oil-based oleogel and tiger nut oil were oleic acid (C18:1), linoleic acid (C18:2) and palmitic acid (C16:0). Tiger nut oil stored for 1 day possessed a monounsaturated fatty acid (MUFA) content level of about 74.17%, polyunsaturated fatty acid (PUFA) content level of about 11.03 and saturated fatty acid (SFA) of 14.76%. After 30 days of storage, the MUFA and PUFA content showed a decrease. This trend also appeared during the storage of sunflower seed oil. Crapiste et al. (1999) [58] studied the changes in the main fatty acid composition of sunflower seed oil during storage and concluded that oleic acid levels increased and linoleic acid levels decreased during storage [58]. Maskan and Karatas (1998) [59] observed a decrease in the percentage of polyunsaturated fatty acids during pistachio storage [59]. A study by Rabadan et al. (2018) [60] also found that the percentage of PUFAs in three nut oils decreased during storage, possibly due to the higher oxidation rate of these fatty acids [60]. There were many branches in the process of oil and fat oxidation, including oligomerization and reorganization. Among them, free radical reaction was one of the main mechanisms of oil autoxidation. Fats in the process of autoxidation, unsaturated fatty acids would react with oxygen molecules in the air to produce peroxides and hydroperoxides free radicals, these peroxides and hydroperoxides were unstable, easy to further pyrolysis and polymerization, to form polymers and low molecular aldehydes, ketones, acids and other new substances. In this process, some unsaturated fatty acids were consumed [61,62].

It could be seen that a certain degree of lipid oxidation occurred during the storage period of tiger nut oil. It was because the saturation degree of fatty acids in the oil was closely related to the oxidation stability of the oil. The oxidation of fats was determined by the unsaturated fatty acids; the higher the degree of unsaturation in the fat molecule, the more pronounced the oxidization occurs, and the saturated fatty acids were the most stable. Interestingly, although the tiger nut oil-based oleogel and tiger nut oil possessed similar trends during the storage period from 1 to 30 days, the extent of oxidation of unsaturated fatty acids was significantly reduced. However, the extent of unsaturated fatty acid oxidation was obviously reduced because of the formation of a stable oleogel structure by xanthan gum and whey proteins, hindering the oxidation rate of oleaginous soybean oil. Therefore, the preparation of tiger nut oil-based oleogel using xanthan gum and whey protein may be a feasible and practical method to slow down the oxidative rancidity of tiger nut oil during storage.

## 4. Conclusions

The focus of this study was on the construction of tiger nut oil-based oleogels with different concentrations of WPI and XG using the emulsion template method. It could be inferred from the emulsion particle size measurements, rheological tests, and microscopic analyses that XG alone could not form HIPE, and particles formed by WPI and high-concentration XG seem to be effective stabilizers of HIPE and oleogels. The results of rheological tests, microscopic analysis, oil loss, and oxidative stability measurements showed that the high concentration of WPI had a positive effect on the stability of the oleogels. In addition, the addition of different concentrations of XG promoted the stability of tiger nut oil-based oleogels and increased with an increase in XG concentration, probably due to the gel network structure formed by WPI and XG, which better encapsulated the liquid oil and subsequently led to more stable oleogels and less oil leakage. WPI (2.0%) and XG (>0.3%) could be used to obtain HIPE with good physicochemical and viscoelastic properties, which in turn lead to oleogels with minimal oil loss, viscoelastic and thixotropic recovery, and temperature stability. In summary, the developed tiger nut oil-based oleogel has a unique microstructure, good viscoelasticity, and high oxidative stability. This presents a promising approach for the partial replacement of butter by tiger nut oil-based oleogels, which could reduce the level of trans fatty acids and saturated fatty acids in food and thus improve the diet of the population. The digestive characteristics, fatty acid composition, and specific applications of tiger nut oil-based oleogels in food products (baked goods, processed meat products, cold beverages) compared with butter need further study.

## Figures and Tables

**Table 1:** Distribution of fatty acids in tiger nut oil and oil gel during storage.

Fatty Acid	Tiger Nut Oil		Tiger Nut Oil-Based Oleogel	
	D1 (1 Day)	D30 (30 Day)	D1 (1 Day)	D30 (30 Day)
Tetradecanoic acid (C14:0)	0.0 ± 0.00 ^a^	0.00 ± 0.01 ^a^	0.04 ± 0.01 ^a^	0.02 ± 0.01 ^a^
Cetylic acid (C16:0)	12.39 ± 0.07 ^a^	12.15 ± 0.20 ^a^	12.30 ± 0.10 ^a^	12.27 ± 0.83 ^a^
Octadecanoic acid (C18:0)	1.96 ± 0.07 ^b^	1.74 ± 0.20 ^bc^	1.93 ± 0.11 ^a^	1.79 ± 0.11 ^c^
Oleinic acid (C18:1)	73.99 ± 0.60 ^b^	71.11 ± 0.80 ^b^	73.30 ± 0.19 ^a^	73.19 ± 0.15 ^c^
Linoleic acid (C18:2)	10.87 ± 0.24 ^b^	10.31 ± 0.16 ^b^	10.56 ± 0.13 ^c^	10.54 ± 0.30 ^a^
Linolenic acid (C18:3)	0.16 ± 0.02 ^b^	0.10 ± 0.01 ^c^	0.15 ± 0.04 ^a^	0.13 ± 0.04 ^bc^
Arachidic acid (C20:0)	0.34 ± 0.05 ^a^	0.20 ± 0.06 ^a^	0.35 ± 0.04 ^a^	0.30 ± 0.09 ^a^
Peanut monoenic acid (C20:1)	0.17 ± 0.02 ^a^	0.10 ± 0.02 ^a^	0.16 ± 0.05 ^a^	0.14 ± 0.06 ^a^
Behenic acid (C22:0)	0.15 ± 0.01 ^b^	0.08 ± 0.03 ^a^	0.13 ± 0.01 ^b^	0.10 ± 0.02 ^b^
Saturated fatty acid (SFA)	14.76 ± 0.06 ^b^	15.86 ± 0.32 ^b^	14.68 ± 0.04 ^a^	15.59 ± 0.72 ^ab^
Monounsaturated fatty acid (MUFA)	74.17 ± 0.58 ^b^	70.30 ± 0.80 ^b^	74.50 ± 0.23 ^a^	72.44 ± 0.20 ^c^
Polyunsaturated fatty acid (PUFA)	11.03 ± 0.26 ^b^	10.0 ± 0.16 ^b^	10.78 ± 0.15 ^c^	10.47 ± 0.34 ^a^
Unsaturated fatty acid (UFA)	85.20 ± 0.32 ^a^	80.11 ± 0.85 ^a^	84.35 ± 0.10 ^b^	83.06 ± 0.51 ^a^

Different letters in the same column indicate significant differences (*p* < 0.05).

## Data Availability

Data is contained within the article.
